# An Intradural Extramedullary Enterogenous Cyst in the Conus Medullaris: A Report of a Rare Presentation

**DOI:** 10.7759/cureus.68971

**Published:** 2024-09-09

**Authors:** Alaa N Turkistani, Wareef Alzahrani, Sara Aljohani, Abdulhakim B Jamjoom

**Affiliations:** 1 Neurosurgery, King Faisal Specialist Hospital and Research Centre, Jeddah, SAU; 2 Neurousrgery, National Guard Health Affairs (NGHA), Jeddah, SAU; 3 Infectious Diseases, College of Medicine, King Saud Bin Abdulaziz University for Health Sciences, Jeddah, SAU; 4 Neurosurgery, King Saud Bin Abdulaziz University for Health Sciences, Jeddah, SAU

**Keywords:** congenital abnormalities, enterogenous cyst, neuroenteric cyst, spinal cord, spinal intradural lesion

## Abstract

Intraspinal enterogenous cysts are rare congenital abnormalities that mainly develop in the spinal canal, more commonly in the cervical and thoracic regions, and rarely in the lumbar spine.

We present a case of neurenteric (NE) cyst in the conus medullaris and cauda equina junction at the level of L1 in a patient presenting with a nine-year history of progressive lower limb weakness, paresthesia, and muscle wasting. The patient underwent complete resection of the cyst and had no postoperative complications with marked improvement of paresthesia and some localized pain in the lower back manageable by analgesics.

## Introduction

Neurenteric cysts (NE), also known as intraspinal enterogenous cysts, represent a rare class of congenital abnormalities stemming from errors during embryogenesis. These epithelium-lined structures, originating from the foregut, predominantly develop within the spinal canal, with a higher prevalence in the cervical and thoracic regions and a markedly lower incidence in the lumbar spine [1,2].

Characterized as intradural, benign lesions, NE cysts may manifest symptoms indicative of spinal cord, spinal nerve root, or cranial nerve compression [3,4]. The disease exhibits a slow progression, with a diverse clinical presentation and fluctuating symptomatology. Definitive diagnosis is achieved solely through histological examination of the lesion, and total surgical excision remains the only curative measure [5,6]. Given the rarity of this condition, we present a case involving a patient who experienced symptoms for nine years before receiving a diagnosis of an intraspinal lesion at the junction between the conus medullaris and cauda equina, which was histologically identified as a neurenteric cyst.

## Case presentation

A 41-year-old man presented with a nine-year history of progressive lower back pain that radiates to his left lower limb associated with radiculopathy. Two years after his initial symptoms, he noticed muscle wasting in his left lower limb that started distally and then progressed proximally over time. Almost three months before coming to our clinic, he started to have right lower limb pain and paresthesia. History was negative for bladder and bowel dysfunction.

On examination, the right lower limb had normal muscle bulk, intact motor function in all muscle groups, intact sensory function, and normal reflexes. However, the left lower limb had visible wasting of quadriceps and calf muscles and scored 4/5 in motor function assessment, decreased sensation with no specific dermatome, and hyporeflexia of the patellar and Achilles tendons. Babinski's sign was negative.

**Figure 1:**
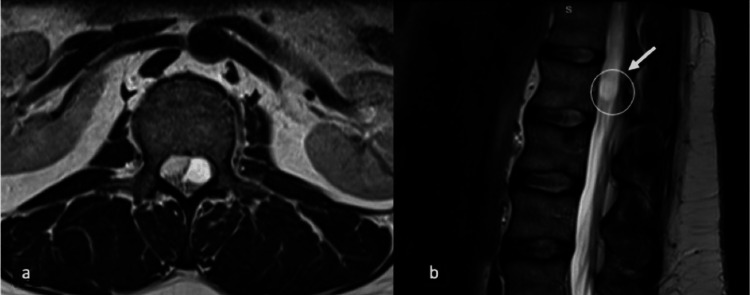
(A) Axial and (B) sagittal T2 weighted MRIs of the lumbar spine show a round extramedullary, intraspinal cystic lesion at the junction between the conus medullaris and cauda equina at L1 level causing mild displacement of the conus medullaris and cauda equina contralateral side. MRI, magnetic resonance image

Magnetic resonance imaging (MRI) of the lumbar spine with gadolinium contrast showed a rounded extramedullary, intraspinal cystic lesion at the level of L1 in between the junction of conus medullaris and cauda equina. The lesion caused mild conus medullaris or cauda equina displacement to the contralateral side without significant compression or enhancement. Other structures of the spine demonstrated no significant associated abnormalities. A preliminary differential based on imaging favored ventriculus terminalis or cystic schwannoma (Figure 1).

During surgery and under general anesthesia, our patient was positioned in a prone position and all pressure areas were padded and a urinary catheter was inserted. A mid-line incision was made from T12 until L1 and subperiosteal dissection was carried out. After exposing the intended area, a total laminectomy of L1 and patrial laminectomy of T12 was carried out. With the help of the neurosurgical microscope, the dura was opened at midline, and a fluid-filled cystic lesion was identified attaching to the nerve root on the left side at the level of T12-L1. We dissected the cyst and cuff of the attachment from the root using micro scissors, and a sample was sent for final histology. The dura was closed using a continuous watertight suturing and then a layer of muscle graft was applied to the surface to secure the seal.

**Figure 2:**
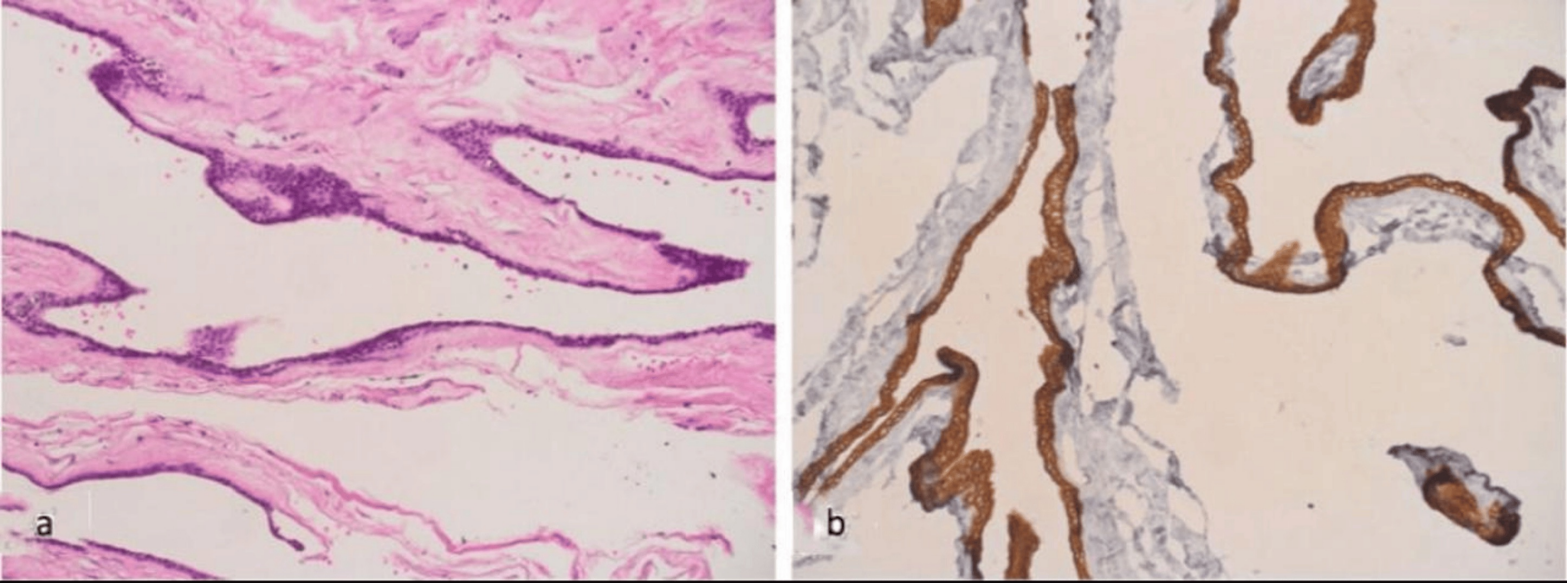
(A) Microscopic examination of the H&E stained section shows a cystic lesion lined by low columnar cuboidal cells. (B) Immunohistochemistry of PAN-cytokeratin highlighting the lining epithelium. H&E, hematoxylin and eosin

During the histological gross examination, a single rounded greyish-white, thin-walled, sac-like fibromembranous soft tissue was seen with measurements of 1 x 0.5 x 0.2 cm. Microscopic examination showed a cystic lesion lined by low columnar to cuboidal cells with no atypia or mitotic figures (Figure 2a). Immunohistochemistry was positive for cytokeratin (Figure 2b) and EMA but negative for S-100, BerEP, and CEA. These results demonstrated that the lesion was an endodermal cyst. Thus, the patient was subsequently diagnosed with an intraspinal enterogenous cyst.

Post-operatively, the patient did not develop new motor or sensory deficits. Lower limb numbness improved, with mild pain localized to the lumbar region, controlled by analgesics. However, the patient still experienced occasional back pain and leg pain. Post-operative MRI showed complete resection of the lesion.

## Discussion

NE cysts, also known as intraspinal enterogenous cysts, represent a rare class of congenital abnormalities stemming from errors during embryogenesis. These epithelium-lined structures, originating from the foregut, predominantly develop within the spinal canal, with a higher prevalence in the cervical and thoracic regions and a markedly lower incidence in the lumbar spine [1,2]. Characterized as intradural, benign lesions, NE cysts may manifest symptoms indicative of spinal cord, spinal nerve root, or cranial nerve compression [3,4]. The disease exhibits a slow progression, with a diverse clinical presentation and fluctuating symptomatology. Definitive diagnosis is achieved solely through histological examination of the lesion, and total surgical excision remains the only curative measure [5,6]. Given the rarity of this condition, we present a case involving a patient who experienced symptoms for nine years before receiving a diagnosis of an intraspinal lesion at the junction between the conus medullaris and cauda equina, which was histologically identified as an NE cyst.

Intradural extramedullary NE cysts of the spine constitute a rare entity, accounting for 0.7-1.3% of spinal tumors. These lesions predominantly occur in the cervical and thoracic regions, with a significantly lower incidence in the lumbar region, as observed in our present case [4,7]. Current understanding posits that NE cysts arise from endodermal origins due to a failure to separate from the ectodermal layer during embryogenesis, a hypothesis supported by the presence of mucin-producing epithelium described in certain reports [8,9]. This notion is further corroborated by the association of NE cysts with vertebral and spinal cord abnormalities [1,10].

Lesions in the cervical and thoracic spine primarily manifest as myelopathic symptoms, whereas radicular symptoms, such as focal weakness, paresthesia, and radicular pain, are more commonly associated with lesions affecting the cervical or lumbar spine. Periodic leakage of fluid content from NE cysts, resulting in volumetric changes, accounts for the fluctuating nature of the symptoms [11]. The variable symptomatology secondary to volume instability often leads to misdiagnosis, with multiple sclerosis being a common erroneous identification [12].

Imaging plays a crucial role in the evaluation of patients presenting with such symptoms. MRI is the optimal imaging modality for this purpose. The most frequently observed radiological feature of NE cysts is a homogenous appearance with isointense signaling in T1-weighted sequences and hyperintense signaling in T2-weighted sequences, without enhancement following contrast administration [7,13,14]. This radiological profile aligns with the findings of the present case. However, variable appearances have been reported, contingent upon the protein content of the cyst [14].

Owing to the variability in clinical presentation and radiological features, histopathology remains the sole diagnostic method for spinal NE cysts. Histopathological characteristics of NE cysts may resemble those of respiratory or gastrointestinal tissues. Consequently, alternative terminologies, such as endodermal cyst (in reference to its origin) or bronchogenic cyst (indicating the presence of respiratory epithelium), have been employed to describe NE cysts [15]. Surgical intervention, specifically total cyst removal, is the preferred treatment option, aiming to alleviate neural compression and prevent cyst refilling and recurrence [16].

Total resection, when feasible, constitutes a curative treatment for sensory and motor deficits. Nonetheless, surgical-related risks must be considered, as unfavorable outcomes following surgery have been documented. Approximately 11% of cases experience symptom worsening, while 18% report failure to regain premorbid function [17]. However, due to the disease's rarity, long-term follow-up data remain scarce.

## Conclusions

NE cysts are rare congenital lesions that typically develop in the cervical and thoracic segments of the spine, with a markedly lower incidence in the lumbar region. We recommend including it in the differential diagnosis of spinal lesions. Patients commonly present with focal pain, weakness, and paresthesia resulting from nerve compression, which may be intermittent due to cyst volumetric changes. Histopathological examination serves as the exclusive diagnostic test, and total surgical resection represents the optimal treatment modality and the sole curative option.
